# Effect of Internal Microstructure Distribution on Quasi-Static Compression Behavior and Energy Absorption of Hollow Truss Structures

**DOI:** 10.3390/ma13225094

**Published:** 2020-11-12

**Authors:** Huilan Ren, Haiting Shen, Jianguo Ning

**Affiliations:** School of Mechatronical Engineering, Beijing Institute of Technology, Beijing 100081, China; huilanren@bit.edu.cn (H.R.); ldshenhaiting@126.com (H.S.)

**Keywords:** hollow truss structure, internal microstructure distribution, compressive behavior, energy absorption capacity

## Abstract

In this work, hollow truss structures with different internal microstructure distributions, i.e., basic hollow truss structure (specimen HT), hollow truss structure with internal microstructure at joints (specimen HTSJ), and hollow truss structure with internal microstructure on tube walls (specimen HTSW), were designed and manufactured using a selective laser melting technique. The effect of internal microstructure distribution on quasi-static compressive behavior and energy absorption was investigated by experimental tests and numerical simulations. The experimental results show that compressive strength and specific compressive strength of specimen HTSW increase by nearly 50% and 14% compared to specimen HT, and its energy absorption per volume and mass also increase by 52% and 15% at a strain of 0.5, respectively. However, the parameters of specimen HTSJ exhibit limited improvement or even a decrease in different degrees in comparison to specimen HT. The numerical simulation indicates that internal microstructures change the bearing capacity and structural weaknesses of the cells, resulting in the different mechanical properties and energy absorptions of the specimens. Based on the internal microstructure design in this study, adding microstructures into the internal weaknesses of the cells parallel to the loading direction is an effective way to improve the compressive properties, energy absorption and compressive stability of hollow truss structures.

## 1. Introduction

Compared with solid structures, porous structures have a significant advantage in terms of specific stiffness/strength and specific surface area resulting in excellent energy absorption capacity, thermal management and acoustic insulation. Thus, they are widely used in many engineering applications as energy absorbers, lightweight structural components, thermal transfer/shields and biological bone grafts [1,2,3,4]. Although matrix material and relative density are important factors to determine the properties of porous structures, diverse cellular structures are the main reason why porous structures become integrated structure-function engineering materials. Hence, it is necessary to investigate the effect of porous structure on properties.

Conventional porous structures contain a large number of randomly distributed pores, and the shape, size and distribution of the pores are usually irregular. For example, Tane et al. [5] produced porous iron using the continuous-zone-melting method in a pressurized nitrogen and hydrogen atmosphere, and the pore size and location of the corresponding specimen were stochastic. Xia et al. [6] manufactured closed-cell aluminum foams using the melt foam method, and the cross-section of the cell shape was a polygon with uncertain sides. Previous research has shown that stochastic pores are not conducive to obtaining high compressive properties for porous structures due to higher stress concentration and earlier local failures, while ordered porous structures exhibit relatively uniform stress distribution and excellent mechanical properties in the family of porous structures [7,8]. Hence, more and more researchers are focusing on the study of ordered porous structures.

In recent years, a variety of ordered porous structures can be fabricated with the advent and development of the additive manufacturing (AM) technique, especially the rapid development of selective laser melting (SLM). SLM technology, which is one of the advanced AM processes, is widely used in manufacturing metal porous structures due to its short process cycle and wide molding range [9]. The ordered porous structures with different cells, such as rhombic dodecahedron [10], octahedral [11], diamond [12] cubic [12], body-centered cubic [13], crystal-line lattice [14], and honeycomb [15], have been developed and widely concerned in previous work. These studies mainly focus on the effect of cellular structures on mechanical performance. With AM technology maturing, in addition to the various structural design and mechanical properties, more works starts to investigate how to obtain an ideal structure with excellent compressive properties and energy absorption when they are used as lightweight structure components and energy absorption devices [16,17,18]. At present, most of the porous structure designs that are widely concerned are solid strut structures. However, only a small number of people pay attention to the hollow strut structures, and some studies have shown that hollow strut structures can achieve better compression performance compared to foam and solid strut structures of a similar relative density [19]. For example, Douglas et al. [20] compared hollow and solid truss pyramidal sandwich structures with the same relative density of 2.8%, and results indicated that the peak stress of the hollow truss structures was approximately twice than that of the solid. Evans et al. [21] theoretically studied the energy absorption characteristics of hollow tube microlattice structures and demonstrated that the hollow tube microlattice structures presented promising performance indices due to the higher second area moments of hollow struts. Additionally, Liu et al. [22] further found that the dynamic energy absorption of hollow microlattice structures was significantly higher than the quasi-static. Zhao et al. [23] concluded that the energy absorption of the hollow structures can be significantly enhanced by inertial stabilization, a shock wave effect and strain rate hardening effect under dynamic loading.

Although hollow strut structures present a significant advantage of compression performance in the porous structure family, how to further promote the energy absorption potential of hollow strut structures is still a challenging task. Liu et al. [24] designed the hollow microlattice structures and proposed several strategies to improve the energy absorption indices: (1) tube wall with gradient thickness can generate plastic buckling from top to bottom during compression, thereby avoiding a sudden collapse of hollow struts and improving energy absorption capacity; (2) using tube with gradient variation of radius makes the plastic deformation propagate progressively and raises the energy absorption density by over 40%; (3) hollow microlattice with water filler strengthens the microlattice via circumference expansion and suppression local wrinkles increases the volume energy absorption density by more than 100%.

In this work, an innovative design method was introduced to improve the compressive properties and energy absorption of hollow strut structures. Namely, the interior of hollow struts was redesigned through adding a series of tubular internal microstructures to strengthen resistance to external load, which was expected to obtain an effective way to improve compression performance from within the hollow struts. Thereafter, a series of hollow truss structures with different internal structures were designed and manufactured using the selective laser melting technique. Quasi-static compression experiments were carried out so as to investigate how the mechanical behavior and energy absorption of the hollow truss structures change with the different internal microstructures. This study was expected to demonstrate that a reasonable addition of internal microstructures was conducive to improving the compressive properties and energy absorption capability for hollow truss structures.

## 2. Materials and Methods

### 2.1. Design of Hollow Truss Structures

**Figure 1:**
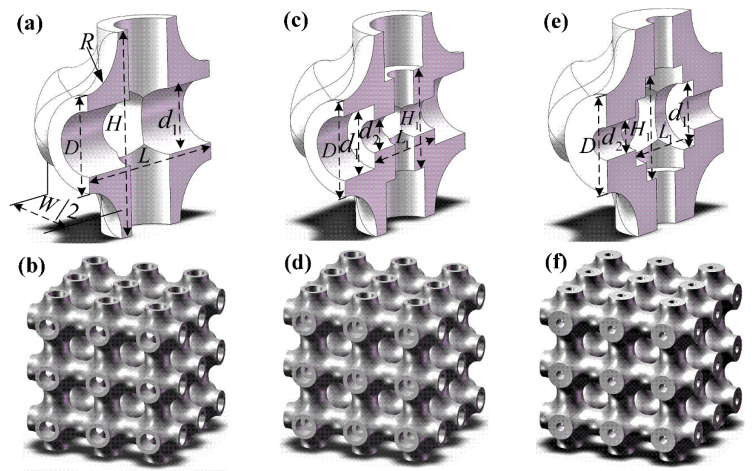
Semi-cellular models and corresponding design specimens: (**a**) semi-cellular model of hollow truss, (**b**) basic hollow truss structure (specimen HT), (**c**) semi-cellular model with tubular microstructures at joint, (**d**) hollow truss structure with internal microstructures at joints (specimen HTSJ), (**e**) semi-cellular model with tubular microstructures on tube walls, and (**f**) hollow truss structure with internal microstructures on tube walls (specimen HTSW).

Hollow truss structures were modeled by the Solidworks software (2014sp 1.0, Dassault aircraft company, Paris, France) in this work. Axisymmetric semi-unit cells and full-size specimens were exhibited in Figure 1 to clearly show the structural characteristics. Each cell was repeated along the normal coordinate axis, and three-unit cells in each axis were patterned to obtain a full-size truss structure. It can be observed that the truss structures were generated using the network struts. Meanwhile, the joint regions were strengthened by chamfers to avoid easy breakage in these positions.

The first group of truss structures was composed using hollow struts as shown in Figure 1a,b, and the second group of truss structures was constructed by hollow struts with orthogonal tubular microstructures at the joints, which are displayed in Figure 1c,d. The third group of truss structures was strengthened by tubular microstructures on the tube walls of hollow struts, and the corresponding structural characteristics are presented in Figure 1e,f. Herein, it should be pointed out that the tubular microstructure positions of the second and third groups of specimens were completely complementary. For the convenient record, the three groups of specimens (basic hollow truss structure, hollow truss structure with internal microstructure at joints, and hollow truss structure with internal microstructure on tube walls) were named in sequence: specimen HT, specimen HTSJ and specimen HTSW, which are exhibited in Figure 1b,d,f, respectively. The dimension of unit cells was $5\times 5\times 5\;{\mathrm{mm}}^{3}$ (L=W=H=5 mm), and radius R of the chamfer is 1.3 mm. At the same time, the internal geometrical parameters of the cells are listed in Table 1.

### 2.2. Fabrication and Tests

The designed hollow truss structures were fabricated using Ti-6Al-4V powder in the present study. The Ti-6Al-4V powder was grade 23 with spherical particle sizes of 20–63 μm. The manufacturing process was carried out by using selective laser melting (FS121M, Farsoon High-technology company, Changsha, China). The approach most utilized for describing the manufacturing process is though the volumetric energy density (E), which is expressed as follows:

(1) $$E=\frac{P}{v\cdot h\cdot t}$$

where P, v, h, and t represent laser power, scanning speed, hatch spacing, and layer thickness, respectively. The detailed processing parameters applied to the present manufacturing process are listed in Table 2. Three duplicates were prepared for the design of each group. Table 3 lists the measured parameters (ρ) of the printed specimens. The measured densities of the specimens were calculated by dividing the volume of hollow truss structures with the mass of measured specimens. In general, the relative density ($\bar{\rho }$) was determined as follow [13]:

(2) $$\bar{\rho }=\rho /\rho_{0}\times 100\%$$

Here $\rho_{0}$ is the density of the matrix Ti-6Al-4V material (4.35 g/cm^3^).

**Figure 2:**
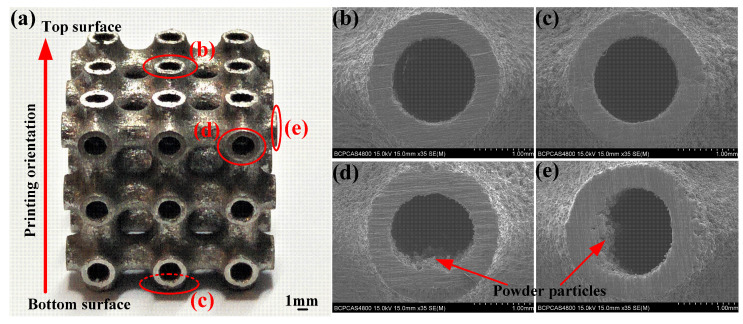
Macro- and micro-observations: (**a**) printed specimen HT, (**b**) top local surface, (**c**) bottom local surface, and (**d**,**e**) side local surfaces.

The structures of specimen HT, which was selected as the representative of all printed specimens, were observed by a Scanning Electron Microscope (SEM, S-4800, Hitachi Limited, Tokyo, Japan). It can be found that the morphologies of the local surface were slightly different, as shown in Figure 2. Melted powder adhesion was clearly observed in the structural outer surface, and, especially, the adhesion of powder particles on the side surfaces was more serious than the top surface and the bottom surface. In fact, the adhesion of powder affects the dimensional accuracy of fabricated structures [25]. In this work, to minimize the effect of adhesion particles on mechanical responses, all specimens were loaded along the top surface and bottom surface, which was also the printing orientation of the specimens. The electron universal testing machine (WDW-300, Changchun Kexin Company, Changchun, China) was employed to perform the compression tests at a strain rate of 10^−3^ s^−1^. Moreover, a high definition digital camera (i-SPEED 3, Olympus corporation, Tokyo, Japan) was utilized to record the deformation behavior of the specimens during the compression.

The following compressive properties of hollow truss structures were determined according to ISO13314-2011: quasi-elastic gradient (E), yield strength ($\sigma_{y}$), first maximum compressive strength ($\sigma_{c}$) and plateau stress ($\sigma_{p}$). The quasi-elastic gradient is the gradient of the straight line in the linear-elastic stage. The yield strength is the 0.2% offset yield stress. The first maximum compressive strength is defined from the initial peak stress in the stress–strain curve. The plateau stress is calculated as the mean stress between 20% and 40% compressive strain.

## 3. Results

**Figure 3:**
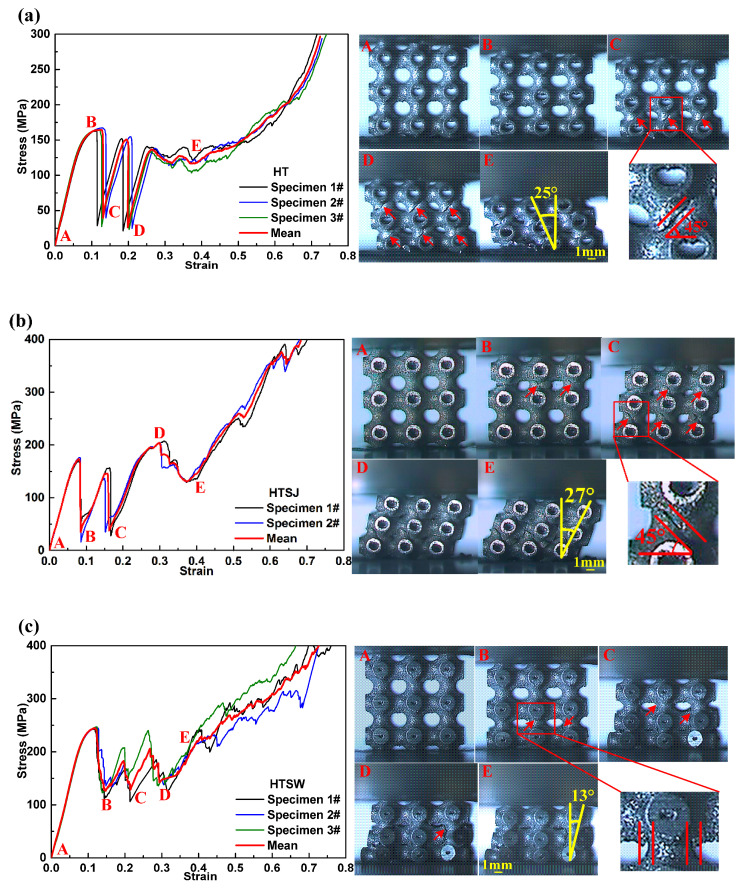
The stress–strain curves and deformation characteristics of the different specimens (**a**) specimen HT, (**b**) specimen HTSJ and (**c**) specimen HTSW.

Compression tests were performed to investigate the compressive properties and deformation behavior of the hollow truss structures. The engineering stress–strain curves and the corresponding deformation process are exhibited in Figure 3. In terms of specimen HT, the stress–strain curve exhibits typical three-stage characteristics. The first stage is the linear-elastic stage where the stress increases almost linearly with increasing strain, and the specimen is initially deformed by elastic buckling of vertical struts (Figure 3(a-A)). Then, the curve reaches yield strength and slowly increases to the first maximum compressive strength. At the same time, plastic yield occurs in the vertical struts of the specimen (Figure 3(a-B)). Afterwards, the second stage is initiated following a post-yield softening up to the onset of densification, and this stage known as the plateau stage and is accompanied by significant stress fluctuations. In this stage, each stress drop corresponds to the collapse of a random pore layer. For example, when the compressive strain is 13% (Figure 3(a-C)), specimen HT exhibits a brittle fracture in the corresponding pore layers. Subsequently, the rest of the structure supports load until the strain approaches 20% (Figure 3(a-D)), and the next collapse layer is crushed. The collapse mode is seen as the failure mode of layer-by-layer manner. At last, the compressive curve enters into the densification stage where the stress starts to increase exponentially. Meanwhile, the specimen is gradually forced into self-contact resulting in the porous structure being squeezed into bulk solids.

Similar compressive characteristics are presented in the loading process of specimens HTSJ and HTSW. However, there are still some differences between the specimens. (i) Specimen HTSW exhibits a remarkably higher yield strength and first maximum compressive strength (Figure 3c). Meanwhile, the plateau stress of specimen HTSW shows relatively stable fluctuations compared to specimens HT and HTSJ. (ii) The collapse positions of specimen HT and HTSJ are located in the middle of struts (Figure 3(a-C,b-C)), but specimen HTSW is collapsed in joint regions of truss structures (Figure 3(c-B)). (iii) The fracture surfaces of the collapsed struts for specimens HT and HTSJ show an angle of 45° from the loading direction. In contrast, the fracture of specimen HTSW is nearly parallel to the loading direction. (iv) The collapse layers of specimen HT and HTSJ occur in horizontal relative movement during the collapse, and the inclinations of the deformed specimens are 25° and 27° at a strain of 0.4 (Figure 3(a-E) and Figure 3(b-E)), respectively. Nevertheless, the collapse layers of specimen HTSW extend around the loading direction (Figure 3(c-E)) and the inclination of specimen HTSW is nearly 13° (nearly half the inclination of the deformed specimens HT and HTSJ), which exhibits the relatively superior structural stability of specimen HTSW during the compression.

## 4. Discussion

### 4.1. Effect of Internal Microstructures on Mechanical Properties

The mechanical parameters of different specimens determined according to ISO13314-2011 are compared in Table 4. Herein, specific parameters are applied by dividing the measured density of truss structures with corresponding mechanical parameters of the specimens to highlight the effect of internal microstructures. Meanwhile, a dimensionless parameter is called the stress drop coefficient (θ), expressed as $\theta =\Delta \sigma /\sigma_{c}$ to characterize the stability of plateau stress [26]. Here, Δσ is the difference value between the first maximum compressive strength and the adjacent valley stress.

It can be seen from Table 4 that specimen HTSW exhibits superior compressive properties. For instance, the yield strength, first maximum compressive strength and plateau stress of specimen HTSW increase by nearly 50% in comparison to specimen HT. At the same time, the increase ratios of specific yield strength, specific first maximum compressive strength and specific plateau stress of specimen HTSW are 14% compared to the corresponding parameters of specimen HT. However, the yield strength and first maximum compressive strength of specimen HTSJ only experience a close to 10% increase compared to specimen HT. Meanwhile, the specific yield strength and specific first maximum compressive strength of specimen HTSJ are even lower than those of specimen HT. This indicates that adding microstructures into tube walls of the hollow truss structure (such as specimen HTSW) is more conducive to improving the compressive properties. Additionally, the stress drop coefficients of the different specimens are compared, and the coefficient of specimen HTSW is significantly lower than the other two sets of specimens. A lower coefficient of stress drop means a better compression stability to some extent. Apparently, specimen HTSW presents a relatively excellent compression stability during the collapse.

**Figure 4:**
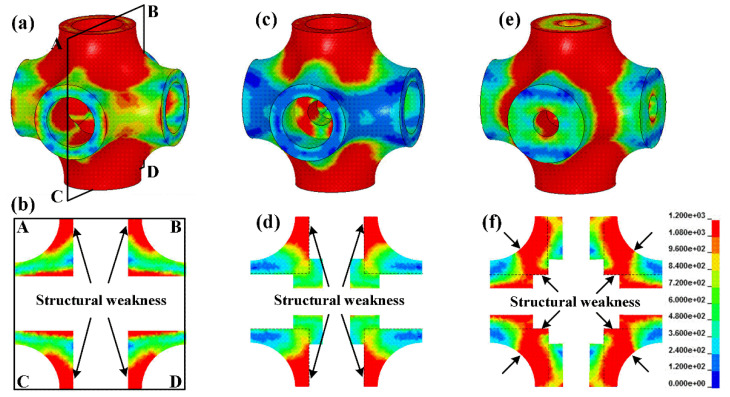
Stress distribution and sectional view ABCD at the strain of 3%: (**a**,**b**) the cell of specimen HT, (**c**,**d**) the cell of specimen HTSJ, and (**e**,**f**) the cell of specimen HTSW.

**Figure 5:**
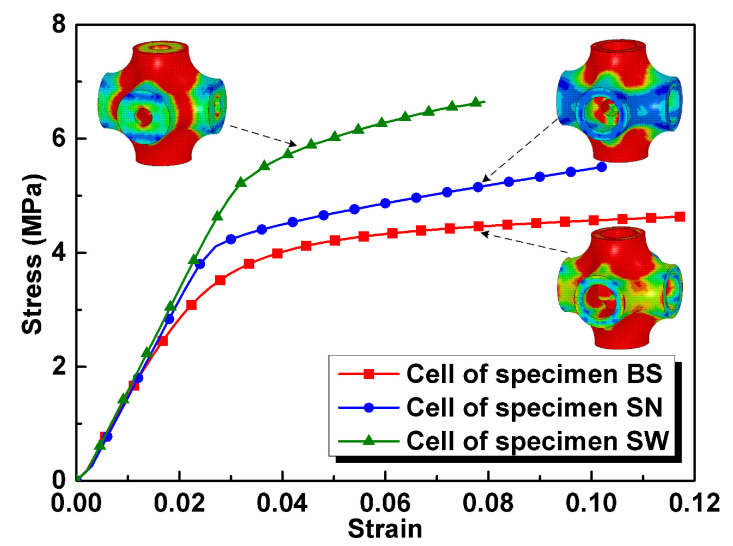
Stress-strain curves of the different cells obtained by numerical simulation.

In order to reveal the reason for the differences in compressive properties of the specimens, LS-DYNA was employed to describe the compressive behavior [27]. Finite element models of unit cells for specimens HT, HTSJ, and HTSW were established, and the elastic-plastic material model was applied to describe the behavior of matrix material. The parameters are as follows: density $\rho =4.35\;g/{\mathrm{cm}}^{3}$, elastic modulus E=110 GPa, yield strength $\sigma_{y}=1080\;\mathrm{MPa}$, and Poisson’s ratio μ=0.3. The cells were loaded from the top surface to bottom surface at a fixed strain rate of 10^−3^ s^−1^. Figure 4 shows the stress distribution of cells and sectional views. It can be seen that the stress concentration of the cells for specimens HT and the HTSJ mainly occurs in the struts along the loading direction, while the stress concentration of the cell for specimen HTSW appears at the joint region. The sectional views clearly show the internal geometry of the different cells and the stress concentration regions are very close to structural weaknesses parallel to the loading direction, as shown in Figure 4b,d,f. It should be noted that the internal microstructures of specimen HTSW reinforce the structural weakness of hollow struts, while the internal microstructures of specimen HTSJ strengthen the non-weak regions of hollow struts. Stress-strain curves of the different cells were extracted from the numerical simulation as shown in Figure 5, and the results show that the compressive stresses were all increased with the addition of microstructures inside the cells. Thus, it can be concluded that adding different internal microstructures changes the structural weaknesses of the original cells and leads to the different bearing capacities of the cells. Moreover, compared to the cell of specimen HTSJ, the compressive stress of the cell for specimen HTSW is higher. This indicates that strengthening the internal weakness of the cells parallel to the loading direction is a more effective way to improve the mechanical properties. Therefore, specimen HTSW presents superior mechanical properties in all specimens due to strengthening the internal weaknesses of the cells.

### 4.2. Effect of Internal Microstructures on Deformation Failure

Although the specimens exhibit the same failure mode in a layer-by-layer manner during compression, there are still some differences affected by internal microstructures such as collapse position, fracture surface, etc. In order to visually reveal the effect of internal microstructures on deformation failure, a numerical simulation is used to describe the collapse process. Herein, maximum shear strain failure criterion is applied, which is set to 0.15 total shear strain. The collapse process of local cells is only simulated, which is enough to reveal the failure characteristics of hollow truss structures.

**Figure 6:**
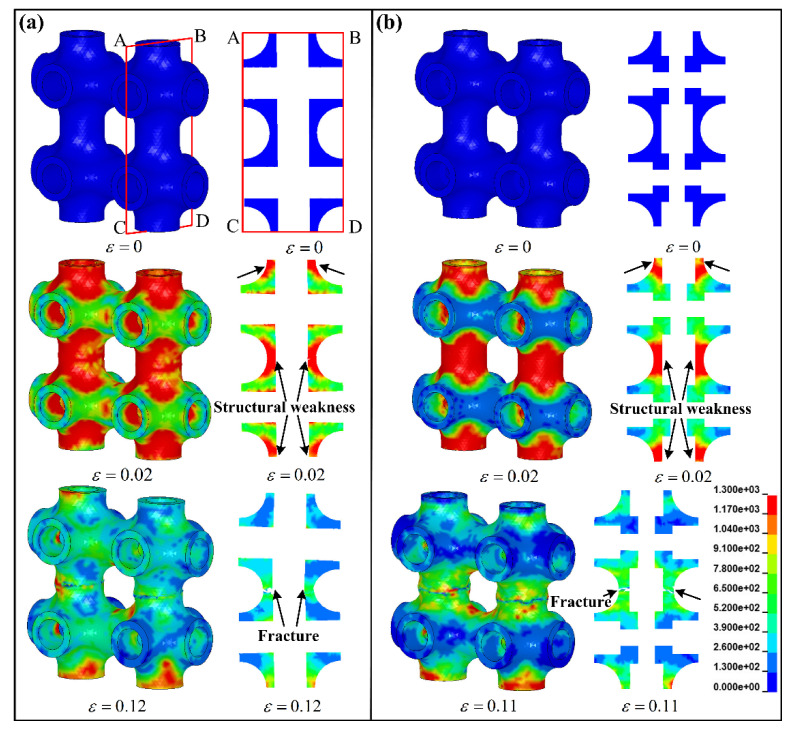
Stress distribution and deformation state predicated by numerical simulation: (**a**) the local cells of specimen HT and (**b**) the local cells of specimen HTSJ.

Figure 6 exhibits the stress distribution and deformation state of the local cells for specimens HT and HTSJ to reveal the effect of internal microstructures on collapse positions. It can be vividly seen that the hollow struts parallel to the loading direction are the main bearing microstructures based to the stress distribution, which can be clearly observed at the strain of 0.02. Then, the hollow struts between the upper and lower cells collapsed in the middle of struts as the strain increased to 0.12. In order to fully reveal the failure characteristics, a sectional view ABCD of the local cells was extracted. It can be observed that the stress concentration of specimen HT is obvious in the hollow struts parallel to the loading direction. The stress concentration regions also correspond to the structural weaknesses of the cells, which are marked by the black arrow in Figure 6a. Afterwards, the collapse position appears in the structural weaknesses. The internal microstructures of specimen HTSJ reinforce the joint regions (non-weak regions of the cell), and the stress concentration also occurs in the hollow struts as shown in Figure 6b. From the failure mechanism of specimens HT and HTSJ, it can be concluded that the structural weaknesses (the middle of hollow struts) parallel to the loading direction cause the stress concentration and lead to the collapse of hollow struts. Actually, the collapse position is largely determined by the structural weakness in the loading direction.

**Figure 7:**
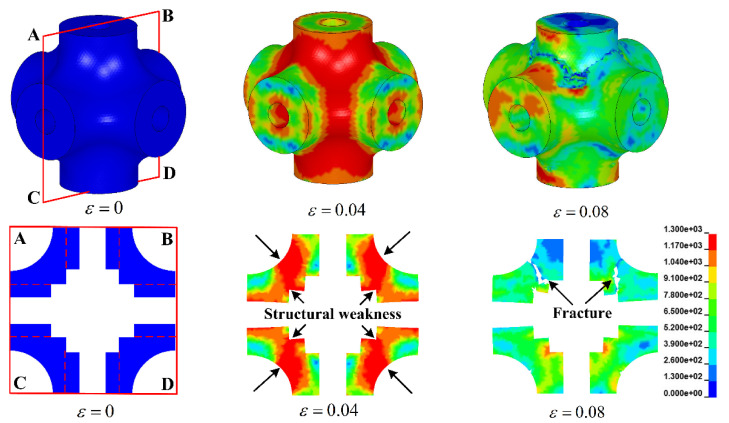
Stress distribution and deformation evolution of unit cell for specimen HTSW.

Figure 7 displays the deformation evolution of the unit cell for specimen HTSW. It intuitively shows that the stress concentration mainly occurs at the joint region of the cell, which can be clearly observed at the strain of 0.04. Compared to the hollow struts of specimen HT, the hollow struts of specimen HTSW are strengthened by internal microstructures. The joint region of specimen HTSW becomes the structural weakness, and the stress concentration mainly occurs in the region marked by the black arrow in Figure 7. With the compressive strain increasing, the joint region gradually collapses. Then, the hollow strut inserts into the abdomen of the joint region, and the collapsed cell extends around the loading direction. Therefore, it can be summarized that the failure mechanism of hollow truss structures is closely related to the structural weaknesses parallel to the loading direction. Different internal microstructures change the structural weaknesses inside the cells, cause the different stress concentrations and affect the collapse position of truss structures. In fact, the collapse of the cell around the loading direction is conducive to obtaining better compressive stability of the specimens during the collapse, which can be confirmed by the aforementioned stress drop coefficient.

Moreover, the internal microstructures affect the characteristics of the fracture surfaces of the specimens. The fracture surfaces of the struts for specimens HT and HTSJ show the angle of 45° from the loading direction, while the fracture surface of specimen HTSW is almost parallel to the loading direction. The fracture surface with a 45° angle of inclination can be attributed to the development of maximum shear stress, which is oriented diagonally to the direction of compression [28]. The failure mechanism can be revealed from the plastic deformation of larger grains. The larger grains within the material deform with a rotation that gives rise to localized softening and is propagated along the diagonal surface [28,29]. It should be pointed out that the fracture surface of the simulated struts shown in Figure 6 is a failure to describe the 45° shear angle due to grid interference, but this does not affect revealing the failure characteristics of the cells.

### 4.3. Effect of Internal Microstructures on Energy Absorption

The effect of internal microstructures on the energy absorption of a hollow truss structure is investigated in this section. It is expected that a more ideal energy-absorbing structure will be obtained through the internal design of hollow truss structures. Here, the energy absorption is determined as [23]:

(3) $$W_{v}=\int_{0}^{\varepsilon }\sigma \left(\varepsilon \right)d\varepsilon$$

(4) $$W_{m}=\frac{\int_{0}^{\varepsilon }\sigma \left(\varepsilon \right)d\varepsilon }{\rho }$$

where $W_{v}$ and $W_{m}$ are the energy absorption capacity (energy absorption per unit volume) and specific energy absorption (energy absorption per unit mass), respectively. ε is the specified strain and σ is the compressive stress. Besides, energy absorption efficiency is also investigated to evaluate the energy absorption behavior which can be expressed as [30]:

(5) $$\eta =\frac{\int_{0}^{\varepsilon }\sigma \left(\varepsilon \right)d\varepsilon }{\sigma_{\max}\varepsilon }$$

where $\sigma_{\max}$ is the maximum stress before the specified strain.

**Figure 8:**
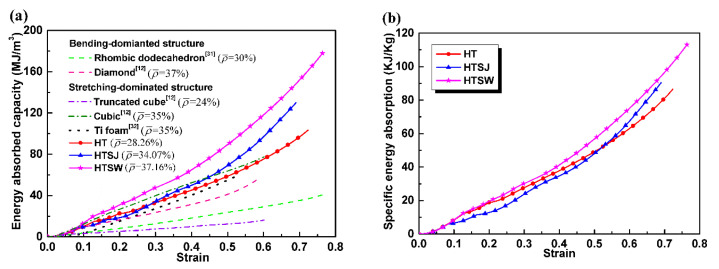
Energy absorbed at different strains: (**a**) energy absorption per unit volume and (**b**) energy absorption per unit mass.

Figure 8 presents the energy absorption capacity and specific energy absorption at different strains. Figure 8a shows that specimen HTSW has an excellent energy absorption capacity compared to specimens HT and HTSJ. For example, the energy absorption capacity of specimen HTSW increases that of specimen HT by 52% and that of specimen HTSJ by 30% at a strain of 0.5, respectively. The reason is that specimen HTSW strengthens the structural weaknesses of specimen HT, improves the compressive stability of plateau stress, and so significantly increases the energy absorption capacity. This indicates that strengthening internal weaknesses of truss structures are conducive to improving energy absorption per unit volume.

Deshpande [2] grouped the porous structures into two categories: stretch-dominated structures and bending-dominated structures. Here, for a comprehensive comparison, the $W_{v}-\varepsilon$ curves of bending-dominated structures (diamond [12], rhombic dodecahedron [31]), stretching-dominated structures (cubic [12] and truncated cuboctahedron [12]) and titanium foam [32] were obtained to compare the energy absorption capacity of the hollow truss structures (stretch-dominated structure in this work). It is worth noting that the matrix material of bending-dominated and stretching-dominated structures is all Ti-6Al-4V, and their relative densities range from 24% to 37%. Although the relative density of the diamond structure, cubic structure, titanium foam, specimen HTSJ and HTSW are very close, specimen HTSW exhibits a stronger energy absorption capacity than the other specimens. In fact, this further verifies that strengthening the internal weaknesses of the hollow truss structures does help to improve the energy absorption capacity.

Considering the effect of specimen mass, the specific energy absorption $W_{m}$ of all specimens is presented in Figure 8b. The $W_{m}-\varepsilon$ curves show that specimen HTSW has the highest specific energy absorption during compression. For example, the energy absorption per unit mass of specimen HTSW increases that of specimen HT by 15%, and that of specimen HTSJ by 18% at the strain of 0.5. Specimen HTSW reinforces the structural weaknesses of the hollow struts, improves the stability of plateau stress, and increases the energy absorption capacity per unit mass. This reveals that strengthening the internal weaknesses of the hollow truss structure is also good for improving energy absorption per unit mass.

**Figure 9:**
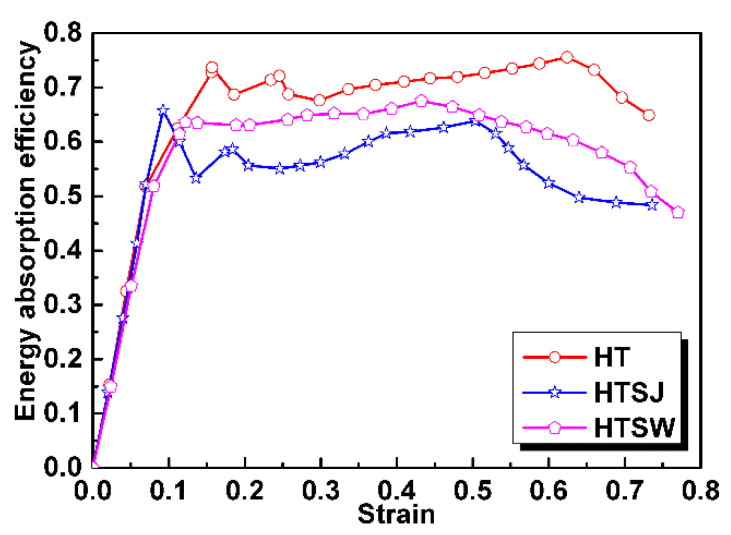
The energy absorption efficiency of the specimens.

Figure 9 presents the η−ε curve characteristics of the specimens including the rising stage, the stable stage and the declining stage. In the rising stage of the curve, the energy absorption efficiency increases rapidly. Next, a relatively steady stage of the energy absorption efficiency within a wide strain range, which is related to the plateau stage of the compressive stress–strain curve, is found. At the declining stage, the energy absorption efficiency gradually decreases. Specimen HT has the most superior energy absorption efficiency including the highest efficiency peak and later decay behavior, resulting from its relatively stable plateau stress and wide strain range. Although the energy absorption efficiency of specimen HTSW takes second place, its η−ε curve has minimal fluctuation over the entire strain range, which shows that specimen HTSW presents the best stability during compression. This shows to some extent that strengthening the internal weaknesses of the hollow truss structure is beneficial to improve compressive stability in the energy absorption process.

## 5. Conclusions

In this work, hollow truss structures with different internal microstructure distributions were successfully fabricated using the selective laser melting technique. The effect of internal microstructure distribution on compressive behavior and energy absorption was investigated. Conclusions were drawn as follows:

(1) When the internal microstructures are distributed in the structural weaknesses of the cells parallel to the loading direction, the compressive strength and specific compressive strength of basic hollow truss structures can be increased by nearly 50% and 14%, respectively. However, when the non-weak regions of the cells are reinforced by internal microstructures, the compressive properties of the specimen exhibit limited improvement or even a decrease in different degrees.
(2) By analyzing the failure characteristics, it can be found that internal microstructures change the structural weaknesses inside the cells, cause the different stress distributions and lead to the different collapse positions of hollow truss structures. Besides, the specimens that collapse at joint regions exhibit more excellent compressive stability during the collapse.
(3) Strengthening the structural weaknesses inside the cells can improve the bearing capacity and the compressive stability of hollow truss structures, so it is conducive to increasing the energy absorption per unit volume and mass.
(4) Adding microstructures into the internal weaknesses of the cells parallel to loading direction is an effective way to improve the compressive properties, energy absorption and compressive stability of hollow truss structures.

## Figures and Tables

**Table 1:** Details of design parameters of the specimens.

Specimen	D (mm)	$d_{1}\;\left(mm\right)$	$d_{2\;}\left(mm\right)$	$H_{1}\;\left(mm\right)$	$L_{1}(\mathrm{mm})$
HT	2.4	1.6	-	-	-
HTSJ	2.4	1.6	0.8	2.5	2.5
HTSW	2.4	1.6	0.8	2.5	2.5

Note: structural parameters (D, d_1_, d_2_, H_1_, L_1_) were marked in Figure 1a,c,e.

**Table 2:** The process parameters used for manufacturing hollow truss structures.

Process Parameters	Values
Laser power (W)	190
Scanning speed (mm/s)	900
Hatch spacing (μm)	100
Layer thickness (μm)	30
Volumetric energy density ($J\cdot {\mathrm{mm}}^{-3}$)	70

**Table 3:** Measured parameters of the printed specimens.

Specimen	$Volume\;\left(mm^{3}\right)$	Mass (g)	$\mathrm{Density}\;\rho \left(g/cm^{3}\right)$	$\mathrm{Relative}\;\mathrm{Density}\;\bar{\rho }$ (%)
HT	15.12×15.07×15.08	4.03±0.16	1.19±0.04	28.26±0.66
HTSJ	15.10×15.18×15.06	4.85±0.23	1.43±0.07	34.07±0.81
HTSW	15.19×15.15×15.07	5.31±0.31	1.57±0.03	37.16±0.78

**Table 4:** Mechanical parameters of the different specimens.

Specimen	E	E/ρ	$\sigma_{y}$	$\sigma_{y}/\rho$	$\sigma_{c}$	$\sigma_{c}/\rho$	$\sigma_{p}$	$\sigma_{p}/\rho$	θ
HT-1#	2137.25	1789.99	128.10	107.29	162.57	136.16	124.35	104.15	0.82
HT-2#	2233.46	1870.57	131.02	109.73	167.52	140.30	113.45	95.02	0.76
HT-3#	2330.63	1951.95	135.18	113.22	163.68	137.09	107.15	89.74	0.83
Average	2237.50	1873.95	131.72	110.31	163.59	137.00	112.88	94.51	0.78
Stand. dev.	93.13	78.00	3.46	2.91	3.93	3.30	5.73	9.64	0.05
HTSJ-1#	2569.43	1788.05	145.85	101.50	170.00	118.30	161.45	112.35	0.79
HTSJ-2#	2507.29	1751.77	151.08	105.14	176.09	122.54	160.95	112.00	0.91
Average	2518.60	1745.02	149.21	103.83	173.11	120.47	161.31	112.25	0.82
Stand. dev.	51.37	43.03	3.36	1.31	2.98	2.07	0.36	0.25	0.09
HTSW-1#	2830.74	1799.58	199.33	126.72	242.37	154.08	161.95	102.96	0.54
HTSW-2#	2643.13	1680.31	193.29	122.88	244.38	155.36	171.00	108.71	0.44
HTSW-3#	2641.07	1679.00	193.02	122.71	247.12	157.10	180.00	114.43	0.50
Average	2701.02	1717.11	197.57	125.60	244.61	155.51	169.87	107.99	0.47
Stand. dev.	129.72	82.47	4.55	2.89	2.51	1.59	−10.13	6.44	0.07

Note: the units of parameters and specific parameters are MPa and MPa$\cdot \;g^{-1}\cdot$ cm^3^, respectively.
